# Bio-Degradable Stents: Primary Experience in a Tertiary Hepatopancreaticobiliary Center in the United Kingdom

**DOI:** 10.7759/cureus.19075

**Published:** 2021-10-27

**Authors:** Osborne P Vaz, Shofiq Al-Islam, Zahid A Khan, Neil Wilde, Beverley Lowe, Anna Magilton, Daren A Subar

**Affiliations:** 1 General Surgery, East Lancashire Hospital Trust, Blackburn, GBR; 2 Radiology, East Lancashire Hospital Trust, Blackburn, GBR; 3 Radiodiagnosis, East Lancashire Hospital Trust, Blackburn, GBR; 4 Hepatobiliary and Pancreatic Surgery, East Lancashire Hospital Trust, Blackburn, GBR

**Keywords:** endoscopic retrograde cholangiopancreatography (ercp), bilioenteric anastomosis, percutaneous transhepatic cholangiography, biodegradable stents, benign biliary strictures

## Abstract

Background: Management of benign biliary strictures (BBS) post bilioenteric anastomoses requires a multidisciplinary approach including surgical, radiological, and/or endoscopic input. Patients often need multiple hospital visits for treatment with the long-term possibility of restenosis. Conventionally BBS have been treated with serial percutaneous transhepatic biliary dilatations necessitating repeat procedures for drain exchange or removal. Surgery may become necessary in refractory strictures. In the last decade, there have been increasing reports of the use of biodegradable stents (BDS) in treating biliary strictures mainly to address the need for repeated procedures for drain exchange.

Aim: This study aimed to report the early outcomes in patients with BBS treated with BDS.

Methods: Retrospective analysis of prospectively collected data was performed in patients who had a bilioenteric anastomosis presenting with an anastomotic stricture and were intended to be treated with BDS. The primary endpoints reported were technical success (defined as a successful resolution of stricture on repeat cholangiogram) and clinical success (defined as the absence of repeated cholangitis). Clavien-Dindo (CD) grade of complication was reported.

Results: Twelve patients presented with BBS and nine patients had BDS. Three patients were not considered suitable for BDS due to a non-traversable stricture and had surgery. The male-female ratio was 1:2. There was 100% technical and clinical success with one patient having stent migration not needing intervention. The procedure took an average of 45 min. In seven (77.7%) patients, it was safely performed under local anesthesia with sedation. Two patients preferred general anesthesia. There was no restenosis noted at a median follow-up of 11 months.

Conclusion: The use of BDS in the treatment of BBS is a safe and effective procedure. Longer-term follow-up with multi-institutional reporting on a national database is needed to assess its long-term benefits.

## Introduction

Benign biliary strictures (BBS) post bilioenteric anastomosis are seen in 2.6%-17% of patients [1]. Those due to injuries post laparoscopic cholecystectomy (LC) are seen in 0.3%-0.7% of patients [2-3]. Management requires a multidisciplinary approach including cross abdominal imaging including magnetic resonance cholangiopancreaticography (MRCP) and/or CT. These investigations help delineate the anatomy with respect to location, number of strictures, length, and associated anatomical anomalies including vascular injuries. While there is no standard accepted classification for BBS, the application of the Bismuth classification in BBS may assist in clinical treatment decision making [3]. Historically surgery was the only option but since the 1970s non-operative management is generally now considered the first treatment of choice [4-6]. It is less invasive, has lesser morbidity, and shorter hospital stay when compared to surgery. The success rates are comparable to surgery [6]. However, there is no gold standard in the treatment of BBS. In patients with no prior bilioenteric anastomosis endoscopic retrograde cholangiopancreatography (ERCP) conventionally remains the first intervention of choice [3]. However, in those with altered anatomy due to bilioenteric anastomosis or an endoscopically non-traversable stricture, percutaneous transhepatic biliary drainage (PTBD) is the first intervention of choice [4, 7]. PTBD has been reported to be less invasive and pose a lesser risk than surgery [2, 8]. However, classically PTBD needs repeated procedures to sequentially upsize the drains and serially dilate the stricture which has led to an increasing interest in the use of biodegradable stents (BDS). Placement of BDS has many advantages over PTBD as it removes the need for repeated procedures, improves the quality of life as the patient has no external drainage catheter to manage, and reduces the risk of complications associated with external drainage [9].

## Materials and methods

This is an observational study with retrospective analysis of prospectively collected data at a tertiary hepato-pancreato-biliary (HPB) center in the United Kingdom.

**Figure 1:**
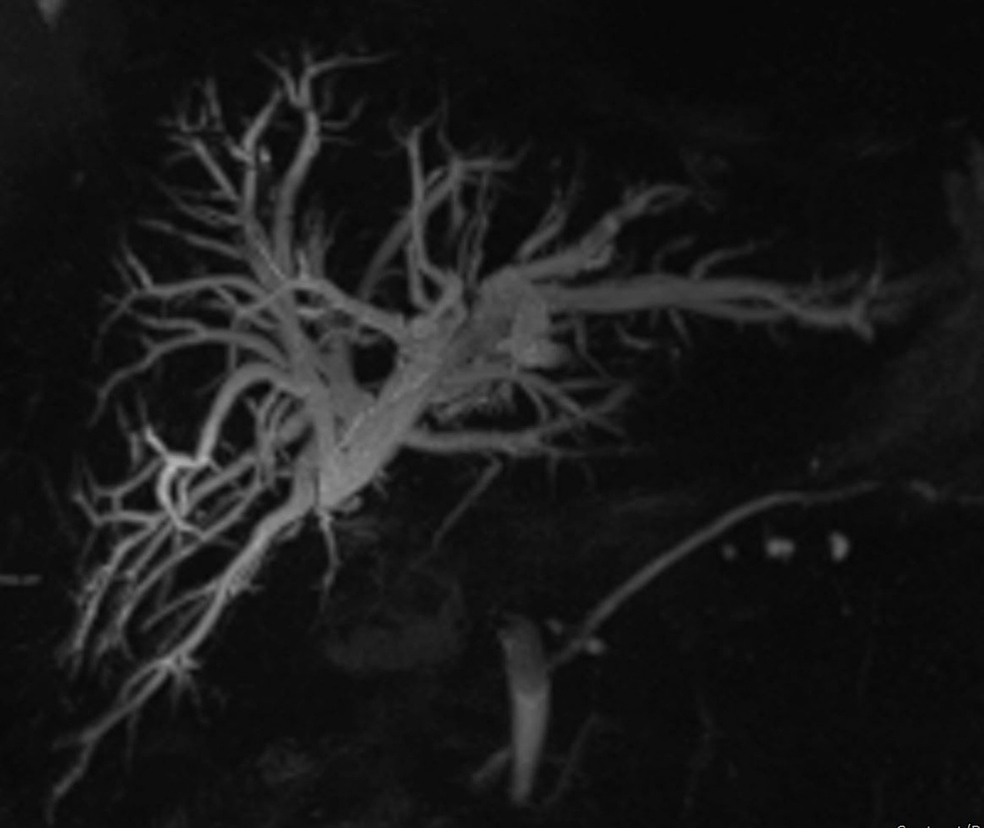
MRCP demonstrating a stricture at the hepaticojejunostomy site. MRCP, magnetic resonance cholangiopancreaticography

All patients who had a request for radiological treatment of a biliary stricture with BDS between January 2017 and June 2020 were recorded on a database. These patients were considered potentially suitable for BDS after a multidisciplinary team discussion. Patient details were acquired from the records of the radiology and surgical departments of the hospital. The details of the procedure were accessed from the procedure notes kept in the patient records and the electronic patient record (EPR) system. Details on follow-up were accessed via the EPR and outpatient clinic notes. The stent used was a polydioxanone stent and all were custom made and procured from SX-ELLA ® (S.R.O, Hradec Kralove, Czech Republic). All stent insertions were done by qualified interventional radiologists. The protocol followed for the patients with biliary stricture was cross-sectional abdominal imaging, followed by a per cutaneous transhepatic cholangiogram with biliary decompression (Figure 1 demonstrating a stricture seen on MRCP).

**Figure 2:**
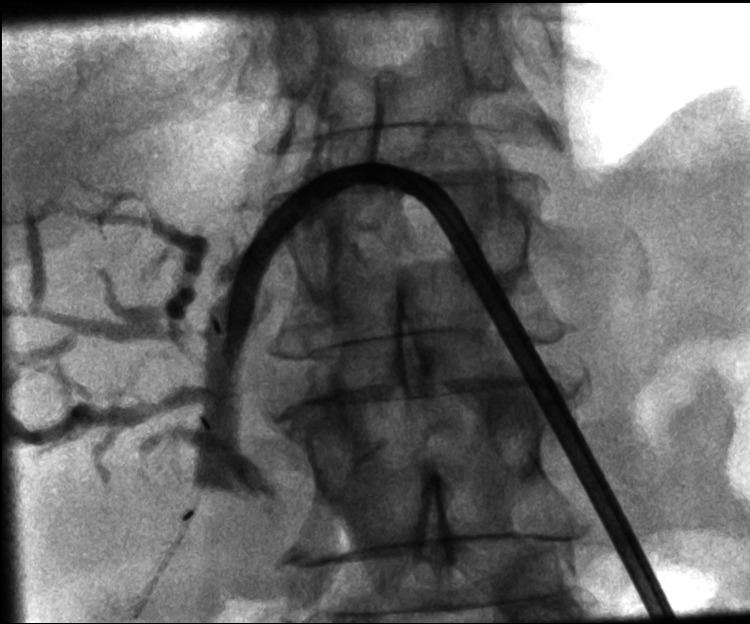
Cholangiogram demonstrating BDS seen with radio-opaque markers with external drain in situ. BDS, biodegradable stents

The patient then underwent serial dilatations with up-sizing of the external drain to 16 Fr. If the stricture persisted, they were considered for insertion of a BDS. The dilated stricture was mapped for dimensions and a custom-made stent was procured from the aforementioned company. The procedure was performed by two interventional radiologists. Post-BDS placement, a check cholangiogram was carried out after seven days (Figure 2 cholangiogram post stent placement).

The external biliary drain was removed if the cholangiogram was satisfactory, and the stent tract filled with Avitene® (BD, Crawley, United Kingdom) for hemostasis, and the chances for bile leak were reduced. Descriptive statistical analysis was done for all patients. Variables included age, gender, primary surgical procedure, number of PTBDs, attempts at BDS placement, technical and clinical success, complications, and follow-up. The complications included bleeding, bile leak, drain displacement, cholangitis, drain obstruction, vascular injury, biliary peritonitis, injury to intra-abdominal viscera, and pleural complications. The Clavien-Dindo (CD) classification was used to categorize the grade of complication.

## Results

There were 12 patients identified with BBS, 11 of whom had bilioenteric anastomosis. One patient had a common hepatic duct stricture post-LC. Three patients did not qualify for a BDS. In these three patients, the bilioenteric stricture was refractory to PTBD and the stricture could not be traversed radiologically. All three patients had a bile duct injury post-LC, two of whom had undergone a hepaticojejunostomy (HJ) and had a HJ stricture. All three underwent successful redo HJs with no post-operative complications to date (Table 1).

**Table 1:** Etiology of benign biliary strictures in the study group. NET, neuroendocrine tumor; HJ, hepaticojejunostomy; CBD, common bile duct; IPMN, intra pancreatic mucinous neoplasm; CHD, common hepatic duct

Patient	Age	Sex	Primary procedure	Stricture location	Definitive procedure
1	77	M	Whipple's procedure for NET	HJ	BDS
2	62	F	HJ for Mirizzi's	HJ	BDS
3	53	F	HJ for CBD injury	HJ	BDS
4	76	F	Whipple's procedure for ampullary carcinoma	HJ	BDS
5	65	F	HJ for CBD injury	HJ	BDS
6	32	M	HJ for CBD injury	HJ	BDS
7	53	F	HJ for CBD injury	HJ	BDS
8	67	M	Whipple's procedure for IPMN	HJ	BDS
9	70	F	HJ post right hepatectomy for colorectal liver metastases	HJ	BDS
10	60	F	HJ for CBD injury	HJ	REDO HJ
11	74	F	HJ for CBD injury	HJ	REDO HJ
12	45	F	Benign biliary stricture post laparoscopic cholecystectomy	CHD	HJ

Nine patients had BDS with a male-female ratio of 1:2. The median age was 65 years (range 32-77 years). All nine (100%) had undergone a HJ in the past which had stenosed. Three (33.3%) had a Whipple’s procedure for pancreatic malignancies, one (11.1%) had a reconstruction after right hepatectomy for colorectal liver metastases with Roux en Y reconstruction. The bile duct was not involved with malignancy in any of these patients and there was no evidence of disease recurrence at the time of BDS insertion. Four (44.4%) patients had a CBD injury post-LC and one (11.1%) had bilioenteric reconstruction post-surgery for Mirizzi’s syndrome (Table 1). The average time to development of anastomotic stenosis was 28.1 months from primary repair (range 4-160). The median number of dilatations needed prior to insertion of the BDS was three (range 2-6). The median time taken in weeks for stent procurement and insertion from the last PTBD was 11 weeks (range 6.5-25.5). Seven (77.7%) of the stent placements were carried out as day case procedures and two (22.2%) had a 24 h stay. Local anesthesia and sedation were used in seven (77.7%) of the patients whilst two (22.2%) needed general anesthesia. The procedure took an average of 45 min. Technical and clinical success was 100%. All patients needed only one BDS placement. One patient had a stent migration that did not require any intervention (CD1). The median follow-up was 11 months (range 4-39 months). No patients required further intervention and no cholangitis and restenosis were noted at follow-up (Table 2).

**Table 2:** Patient demographics, details of stenting, and follow-up. PTBD, percutaneous transhepatic biliary dilatation

	Range	Mean (Median)	Confidence intervals
AGE in years	(32-77)	61.7 (65)	95% (50.8-72.5)
GENDER Male 3(33.3%) Female 6(66.6%)	-		
TIME TO STENOSIS in months	(4-160)	28.1 (18)	95% (-10.4-66.7)
PTBD	(2-6)	(3)	95% (2.5-4.8)
Time to BDS from last PTBD in weeks	(6.5-25.5)	12.8(11)	95% (7.9-17.8)
FOLLOW UP in months	(1.5-39)	18.8(11)	95% (10.9-26.8)

## Discussion

Whilst PTBD with serial dilatations with or without stenting has been the mainstay in BBS post-bilioenteric anastomosis, there has been an emergence in the use of BDS in the past decade [1, 5, 7-16]. The use of BDS was first described in interventional cardiology and thereafter in esophageal and urethral strictures. Recently, there have been increasing reports of its use in the treatment of biliary strictures. This is mainly to address the need for reinterventions to exchange or remove stents and refractory stenosis [3, 17-18]. The first published case report of usage of a biliary BDS was via ERCP and demonstrated technical and clinical success [19]. Thereafter these stents have been used for endoscopic treatment of biliary strictures usually post-cholecystectomy biliary injuries [3, 17] before gaining usage via the percutaneous methods [9, 13]. Manufacturer studies suggest that the radial strength of the stent is maintained for the first five weeks which gradually recedes to two-thirds the initial value at week seven post-insertion, and one-thirds at week nine [7]. The degradation products are not harmful to human tissues. Studies have shown that BDS express proteins that tend to be similar to those observed in intact bile ducts [19-21]. Our study reflected a 100% stent patency rate. This may be due to the fact that it had a short follow-up with a median of 11 months. Larger studies observed a lower patency rate, however, they had a significantly longer term of follow-up. The largest study by de Gregorio et al. suggests that stent patency of 78.9% is observed at 60 months [9]. There were 40(26.6%) patients who had restenosis and occlusion needing intervention. Among these, 18(12%) patients needed a second stent and 22(14.6%) needed definitive operative repair. None of their patients had major complications due to stent placements. Another study by Mauri et al. also demonstrated similar outcomes with stricture recurrence in 18% of patients with an estimated time to re-stricture of 38 months [13]. To the best of our knowledge, this is the largest series in the United Kingdom reported to date. Our study included a small cohort and a short follow-up period. The efficiency of the procedure is limited by the unavailability of a local manufacturing source and the fact that stents are made to order. The stents are off-label and need a special prescription. The process of stent acquisition and insertion took a median of 11 weeks. There is also special permission needed for cost reimbursement which prolongs treatment time. The cost of metal stenting is £2900 and each percutaneous transhepatic cholangiogram (PTC) episode pre- and post-stenting costs £890. Bilioenteric bypass surgery costs £13606. Comparatively the average cost of PTBDs followed by BDS insertion in the patients from this study amounted to £7837. BDS is evidently more cost-effective than surgery. The cost of multiple plastic stenting and metal stentings increases with each additional procedure. Complications due to repetitive procedures also add to the financial burden in fully covered self-expanding metal stenting and multiple plastic stenting. The limitations of our study were a small cohort of patients, short follow-up, and delays due to logistics involved in stent procurement.

## Conclusions

Biodegradable stents insertion via percutaneous transhepatic route can safely be performed as a day case procedure, is cost-effective, and has high success rates. The post-procedural complication rates are minimal. These factors contribute immensely to improving patient outcomes and reducing hospital costs. As it stands, larger multicenter trials are needed. A consensus too needs to be arrived at for standardization of the selection criteria in this small cohort of patients presenting at any single HPB unit.
